# Waist, neck circumferences, waist-to-hip ratio: Which is the best cardiometabolic risk marker in women with severe obesity? The SOON cohort

**DOI:** 10.1371/journal.pone.0206617

**Published:** 2018-11-08

**Authors:** Anne-Laure Borel, Sandrine Coumes, Fabian Reche, Stéphane Ruckly, Jean-Louis Pépin, Renaud Tamisier, Nelly Wion, Catherine Arvieux

**Affiliations:** 1 Hypoxia PathoPhysiology laboratory, INSERM U1042, University Grenoble Alpes, Grenoble, France; 2 Grenoble Alpes University Hospital, Pole DIGIDUNE, nutrition department, Grenoble, France; 3 Grenoble Alpes University Hospital, Pole DIGIDUNE, digestive surgery department, Grenoble, France; 4 ICUREsearch, Paris, France; 5 Grenoble University Hospital, Pole Thorax et Vaisseaux, physiology, sleep and exercise clinic, Grenoble, France; Beijing Key Laboratory of Diabetes Prevention and Research, CHINA

## Abstract

A centralized deposit of adiposity increases the risk of cardiometabolic diseases. Several anthropometric markers can be used to characterize fat distribution. In the case of severe obesity, several markers, such as hip and waist circumference, are prone to measurement error. Conversely, neck circumference is easy to obtain. The aim was to determine the best surrogate marker of obesity-related cardiometabolic diseases from: body mass index (BMI), waist, hip and neck circumferences and waist-to-hip ratio.

**Methods:**

Data from women (n = 305, aged 43 [34; 53] years-old, BMI 44.2 [40.8; 48.2] kg/m^2^) included in the Severe Obesity Outcome Network (SOON) cohort were analyzed (i) to evaluate collinearity between the different anthropometric markers, (ii) to compare the association of markers with hypertension, type 2 diabetes, obstructive sleep apnea syndrome (OSAS) and other cardiometabolic risks.

**Results:**

Hip, waist and neck circumferences correlated with BMI with respectively less collinearity (r = 0.70, r = 0.59 and r = 0.37, respectively, p<0.001) whereas waist-to-hip ratio was not correlated (r = 0.11, p = 0.072). Waist and neck circumferences were significantly associated with hypertension, type 2 diabetes and OSAS in univariate logistic regressions, waist-to-hip ratio with hypertension and type 2 diabetes. Hip circumference was inversely correlated with type 2 diabetes (OR 0.970 (95CI: 0.948; 0.991) p = 0.006). BMI was only linked to OSAS (OR 1.092 (95CI: 1.043; 1.143) p<0.001). Neck circumference was the only marker significantly associated with all cardiometabolic risk markers (HOMA-IR, apnea-hypopnea index, Log Triglycerides/HDL-c, alanin-aminotransferase, aspartate-aminotransferase, gammaglutamyl transpeptidase).

**Conclusions:**

Neck circumference appears the most appropriate anthropometric marker to identify the fat distribution associated with high cardiometabolic risk in women with severe obesity.

## Introduction

Obesity affects physical, psychological and social well-being. Although there is a relationship between obesity and cardiometabolic diseases, the risk of developing such diseases is variable. In the 1950’s studies showed that obesity-related cardiovascular and metabolic comorbidities were associated with a centralized deposit of adiposity [1]. Excess visceral fat is associated with a loss of the capacity to stock excessive calories within subcutaneous adipose tissue, inducing an ectopic fat deposition in the liver, muscles, heart etc. that leads to metabolic syndrome and cardiometabolic diseases [2]. Patients with a central obesity distribution have therefore a higher risk of hypertension, type 2 diabetes, obstructive sleep apnea syndrome (OSAS) and non-alcoholic fatty liver diseases (NAFLD). The risk of cardiovascular diseases is also high [3]. Conversely, obese patients with peripheral or subcutaneous adiposity are usually “metabolically healthy”, and even protected against metabolic diseases because subcutaneous adiposity is both a marker of, and a contributor to, a high level of insulin sensitivity [4, 5].

Waist circumference and waist-to-hip ratio are well established as useful indicators of visceral fat accumulation. These simple anthropometric measurements can detect subjects at risk in the primary care setting. However, waist circumference increases with increasing body weight. Progressively higher median waist values are associated with increasing body mass index (BMI), although for any given BMI value, the variation in waist circumference is considerable. Thus it is difficult to define a single cut-off value for abdominal obesity across a large range of BMIs [6]. Waist-to-hip ratio is not limited in the same way since the collinearity of waist and hip circumferences with BMI is neutralized by dividing one by the other [7]. However, the measurement of waist and hip circumference in patients with severe obesity is hampered by technical issues. The measurement of waist circumference is often complicated by the lumbar lordosis and measurement of hip circumference is difficult due to the abdominal fat apron. Neck circumference is used as an anthropometric marker to detect patients at risk of sleep apnea syndrome [8]; however, this marker also appears to be associated with metabolic disorders [9, 10].

The aim of this study was to compare the level of association of different anthropometric markers with cardiometabolic diseases and cardiometabolic risk markers in women with severe obesity. We sought to determine which of the following parameters was the best surrogate to identify women with a high risk of obesity related diseases: BMI, waist, hip and neck circumferences, and waist-to-hip ratio. In addition, we assessed whether waist-to-height and neck-to-height ratios improved the results obtained with waist and neck circumferences, respectively.

## Materials and methods

The severe obesity outcome network (SOON) cohort recruited adult patients volunteering for bariatric surgery from the 1st of September 2013, at the Grenoble Alpes University Hospital, France. The population studied was mainly of Caucasian people living in France. Inclusion criteria were based on French recommendations; adults aged 18–65 years-old with class III obesity (body mass index (BMI) ≥40 kg/m^2^) or class II obesity (40>BMI ≥35 kg/m^2^) with at least one obesity-related complication (type 2 diabetes, hypertension, OSAS or nonalcoholic steatohepatitis). Exclusion criteria were contraindications to bariatric surgery: pregnancy, psychotic disease and alcohol or drug addictions. The present work analyzed the data from the 305 women included by September 2016. The present study was reviewed and approved by an institutional review board (ethics committee: “Comité de protection des personnes Sud-Est V”, IRB 6705) before the study began. All patients signed informed consent for their inclusion. The SOON cohort is registered on clinical trials (NCT02264431).

### Study design

Observational, transversal study.

### Anthropometric measures

Height, weight, and hip, waist and neck circumference were measured according to standardized procedures at the greater trochanter, iliac crest, and cricoid, respectively [11]. Circumferences were measured with the participant in a standing position using a non-extensible, flexible anthropometric tape.

### Plasma, glucose/insulin homeostasis profile

Women were considered to have type 2 diabetes either based on current medication for diabetes or by HbA1c > 6.5%, fasting plasma glucose above 7 mmol/L over two separate analyses, or according to the results of a 75g oral glucose tolerance test (OGTT) with fasting glucose ≥7 mmol/L or 120 min OGTT-glucose ≥11.1 mmol/L [12]. Insulin resistance was assessed with HOMA-IR index, based on fasting plasma and insulin levels [13].

### Plasma lipid/lipoprotein profile

Plasma HDL-cholesterol, triglycerides, VLDL-cholesterol and LDL-cholesterol were determined according to standardized procedures [14, 15].

### Blood pressure

Hypertension was determined either by current use of anti-hypertensive drugs or by a mean systolic blood pressure above 140 mmHg and/or a diastolic blood pressure above 90 mmHg found during three blood pressure measurements taken 3 min apart on the non-dominant arm, with an appropriate-sized cuff after the patient had been sitting for 5 min [16].

### Sleep apnea syndrome

OSAS was determined either by current use of continuous positive airway pressure therapy or an oral mandibular advancement device, or by the results of nocturnal polygraphy. The recordings were scored according to standardized methods, described in the American Academy of Sleep Medicine (AASM) Manual for Scoring Sleep and Associated Events [17]. Patients with an apnea+hypopnea index (AHI) >30 apnea + hypopnea events per hour were considered to have severe OSAS.

### Functional liver tests

Functional liver enzymes, including alanine aminotransferase (ALT), aspartate aminotransferase (AST), and gammaglutamyl transpeptidase (GT) were evaluated.

### Statistical analysis

Descriptive data are reported as medians [interquartile range] for continuous variables and as numbers (%) for qualitative variables.

Univariate correlations were analyzed using non-parametric Spearman tests to assess collinearity between anthropometric markers. A weighted local regression line (LOESS) was added on graphic representations.

Univariate logistic regressions were performed to address the relationship between anthropometric markers and obesity related diseases (Type 2 diabetes, hypertension, OSAS). The linearity of each variable was tested using a generalized additive model (proc GAM in SAS). One outlier for waist-to-hip ratio conducted to perform a sensitivity analysis for logistic regressions involving waist-to-hip ratio. Multiple logistic regressions were then performed including age, tobacco, alcohol consumption and the presence of depression as covariates in the models.

Univariate correlations were performed using non-parametric Spearman tests to measure the relationship between anthropometric markers and continuous cardiometabolic risk markers (log TG/HDL, HOMA-IR, AST, ALT, GT, AHI).

Models were performed with imputed data according to the FCS logistic regression method, using 10 data sets to deal with missing data (less than 10% in all cases). For patients previously diagnosed and treated for severe OSAS, AHI was missing. Imputation of data for AHI in these patients was performed using the subpopulation of women who were found to have OSAS on nocturnal polygraphy.

The significance level was set at p<0.05. All analyses were performed with the SAS statistical package version 9.4 (SAS Institute, Cary, NC, USA).

## Results

The characteristics of the women included are reported in Table 1. In these mainly middle-aged women, the prevalence of obesity-related diseases was 26.6% for type 2 diabetes, 28.0% for hypertension and 29.9% for severe OSAS.

**Table 1:** Patient characteristics.

	N = 305
Age (years)	43 [34; 53]
Female gender n (%)	305 (100.0)
**Anthropometric markers**	
Body weight (kg)	117 [107; 129]
BMI (kg/m2)	44.2 [40.8; 48.2]
Waist circumference (cm)	123 [115; 130.5]
Hip circumference (cm)	134 [125; 143]
Neck circumference (cm)	40.2 [38; 42]
Waist-to-hip ratio	0.91 [0.85; 0.98]
Neck-to-height	0.25 [0.23; 0.26]
Waist-to-height	0.76 [0.71; 0.81]
**Obesity related diseases**	
Hypertension n (%)	85 (28.0)
Systolic blood pressure (mmHg)	123 [116; 133]
Diastolic blood pressure (mmHg)	70 [63; 77]
Diabetes n (%)	81 (26.6)
HOMA-IR	3.6 [2.3; 6.4]
Sleep apnea syndrome	
Mild n (%)	102 (34.6)
Moderate n (%)	63 (21.4)
Severe n (%)	88 (29.8)
IAH 3% (event/h)	13.8 [7.1; 26]
Number of associated comorbidities*	
0 n (%)	145 (47.5)
1 n (%)	83 (27.2)
2 n (%)	60 (19.7)
3 n (%)	17 (5.6)
**Lipid/lipoprotein profile**	
Triglycerides (mmol/L)	1.5 [1.1; 2]
HDL-c (mmol/L)	1.2 [1; 1.4]
LDL-c (mmol/L)	2.9 [2.4; 3.6]
Log Tg/HDL-c	0.4 [0.1; 0.6]
**Liver functional tests**	
ALT (UI/L)	31 [23.5; 44]
AST (UI/L)	18 [14; 25]
GT (UI/L)	31 [19; 53]

Data are medians (IQR) for continuous variables and n (%) for qualitative variables. Abbreviations: BMI: body mass index; HOMA-IR: HOMA-Index Resistance; Tg: triglycerides; AHI: apnea-hypopnea index; ALT: alanine aminotransferase; AST: aspartate aminotransferase; GT: gammaglutamyl transpeptidase.

*The comorbidities taken into account are hypertension, type 2 diabetes, severe sleep apnea syndrome.

### Collinearity among anthropometric markers

Waist and hip circumferences were highly collinear with BMI (r = 0.59 p<0.001 and r = 0.70 p<0.001, respectively). Neck circumference and BMI were collinear but with a smaller r coefficient (r = 0.37 p<0.001), whereas waist-to-hip ratio was unrelated to BMI (r = 0.11 p = 0.072). Waist-to-height and neck-to-height ratios demonstrated similar results to waist and neck circumferences, respectively (r = 0.61, p<0.001 and r = 0.37, p<0.001, respectively).

Waist circumference and waist-to-hip ratio were also collinear with hip circumference (r = 0.37 and r = 0.53, respectively, p<0.001 for both). Neck circumference was the only anthropometric marker that was collinear with other markers of central adiposity (r = 0.51 with waist circumference and r = 0.34 with waist-to-hip ratio, p<0.001) whereas it was unrelated to hip circumference (r = 0.11, p = 0.084), a marker of subcutaneous adiposity.

### Association between anthropometric markers and obesity related diseases

**Fig 1:**
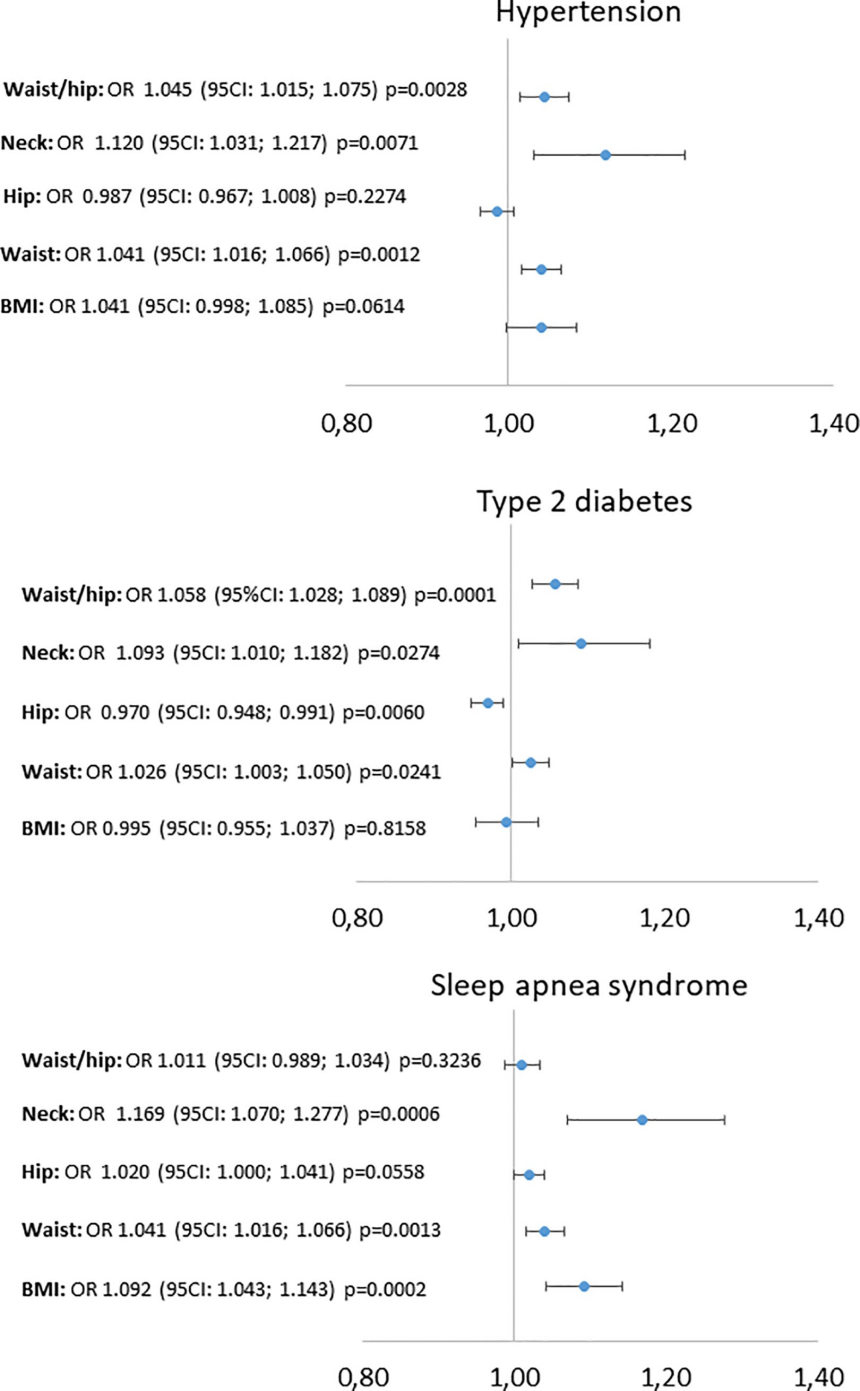
Univariate logistic regressions with anthropometric markers as independent variables and obesity related diseases as dependent variables. Abbreviation: BMI: body mass index; OR: odd ratio; OSAS: Obstructive sleep apnea syndrome.

Univariate logistic regressions are reported in Fig 1. Waist and neck circumferences were significantly associated with hypertension, type 2 diabetes and OSAS. Hip circumference was unrelated to hypertension or OSAS and inversely associated with type 2 diabetes. The logistic regressions with waist-to-hip ratio were influenced by an outlier waist-to-hip ratio of 1.99, due to an extremely large abdominal apron. A sensitivity analysis without this patient led to a significant association of waist-to-hip with hypertension and type 2 diabetes but not with OSAS. These results are reported in Fig 1. BMI was significantly associated with OSAS but not with hypertension or type 2 diabetes. The results for waist-to-height and neck-to-height ratios were similar to those for waist and neck circumferences: OR 1.077 (95CI: 1.035; 1.121), p<0.0001 and OR 1.221 (95CI: 1.073; 1.389), p = 0.002, respectively for association with hypertension; OR 1.054 (95CI: 1.015; 1.093), p = 0.006 and OR 1.179 (95CI: 1.044; 1.331), p = 0.008 for association with diabetes; OR 1.058 (95CI: 1.017; 1.100), p = 0.003 and OR 1.223 (95CI: 1.072; 1.395) p = 0.008 for association with OSAS. The areas under the curve for these logistic regressions are reported in Table 2.

**Table 2:** Areas under the curve of univariate logistic regressions.

	Waist circ.	Hip circ.	Waist-to-hip	Neck circ.	Waist circ.-to-height	Neck circ.-to-height
Hypertension	0,77	0,74	0,78	0,76	0.78	0.76
Type 2 diabetes	0,67	0,67	0,72	0,66	0.68	0.67
OSAS	0,77	0,76	0,75	0,78	0.76	0.77

Abbreviation: OSAS: obstructive sleep apnea; Circ.: circumference.

To test whether such associations were independent of potential covariates, multiple logistic regressions were then performed including age, tobacco, alcohol consumption and the presence of depression as covariates in the models. Results were essentially the same than in univariate logistic analyses. These models are reported in Table 3.

**Table 3:** Multivariable logistic regressions for obesity related diseases.

	B-estimate	Standard error	OR (95%CI)	P values
**Hypertension**				
BMI	0.0431	0.0223	1.044 (0.999;1.091)	0.0534
Neck circumference	0.1215	0.0446	1.129 (1.035; 1.232)	0.0064
Waist circumference	0.0407	0.0129	1.042 (1.015; 1.068)	0.0017
Hip circumference	-0.0103	0.0109	0.990 (0.969; 1.011)	0.3450
Waist/hip raio	0.0385	0.0154	1.039 (1.008; 1.071)	0.0123
Neck circ./height	0.2019	0.0691	1.224 (1.069; 1.401)	0.0035
Waist circ./height	0.0718	0.0212	1.074 (1.031; 1.120)	0.0007
**Type 2 diabetes**				
BMI	-0.00046	0.0221	1.000 (0.957; 1.044)	0.9836
Neck circumference	0.0856	0.0429	1.089 (1.001; 1.185)	0.0462
Waist circumference	0.0260	0.0122	1.026 (1.002; 1.051)	0.0330
Hip circumference	-0.0278	0.0117	0.973 (0.951; 0.995)	0.0171
Waist/hip raio	0.0505	0.0150	1.052 (1.021; 1.083)	0.0008
Neck circ./height	0.1475	0.0664	1.159 (1.018; 1.320)	0.0263
Waist circ./height	0.0495	0.0201	1.051 (1.010; 1.093)	0.0139
**Obstructive sleep apnea syndrome**				
BMI	0.0843	0.0239	1.088 (1.038; 1.140)	0.0004
Neck circumference	0.2015	0.0483	1.223 (1.113; 1.345)	<0.0001
Waist circumference	0.0433	0.0131	1.044 (1.018; 1.071)	0.0009
Hip circumference	0.0170	0.0107	1.017 (0.996; 1.039)	0.1137
Waist/hip raio	0.0140	0.0123	1.014 (0.990; 1.039)	0.2556
Neck circ./height	0.2480	0.0714	1.281 (1.114; 1.474)	0.0005
Waist circ./height	0.0589	0.0210	1.061 (1.018; 1.105)	0.0050

**Abbreviations:** Circ: circumference; BMI: body mass index; CI: confidence interval. Multiple logistic regressions were performed including age, tobacco, alcohol consumption and the presence of depression as covariates in the models.

### Correlations between anthropometric and cardiometabolic risk markers

Spearman correlations are reported in Table 4. Neck circumference (as well as neck-to-height ratio) was the only anthropometric marker that strongly correlated with all cardiometabolic risk parameters, particularly with functional liver enzymes, i.e. ALT, AST and GT. No significant correlation was found between the different anthropometric markers and the LDL-c levels.

**Table 4:** Univariate Spearman correlations between anthropometric parameters and cardiometabolic risk markers.

	BMI	Neck circ.	Waist circ.	Hip circ.	Waist/hip	Neck circ./height	Waist circ./height
**HOMA-IR**	r = 0.07	**r = 0.34**	**r = 0.30**	**r = 0.14**	**r = 0.37**	**r = 0.34**	**r = 0.32**
	p = 0.230	**p<0.001**	**p<0.001**	**p = 0.018**	**p<0.001**	**p<0.001**	**p<0.001**
**AHI**	**r = 0.16**	**r = 0.23**	r = 0.11	r = 0.05	r = 0.05	**r = 0.29**	**r = 0.18**
	**p = 0.010**	**p<0.001**	p = 0.079	p = 0.448	p = 0.431	**p<0.001**	**p = 0.003**
**Log Tg/HDL**	r = 0.00	**r = 0.24**	**r = 0.14**	r = 0.08	**r = 0.22**	**r = 0.22**	**r = 0.15**
	p = 0.966	**p<0.001**	**p = 0.018**	p = 0.167	**p<0.001**	**p<0.001**	**p = 0.008**
**ALT**	r = 0.05	**r = 0.24**	r = 0.08	**r = 0.21**	**r = 0.28**	**r = 0.24**	r = 0.09
	p = 0.351	**p<0.001**	p = 0.147	**p<0.001**	**p<0.001**	**p<0.001**	p = 0.123
**AST**	r = 0.06	**r = 0.14**	r = 0.07	**r = 0.19**	**r = 0.26**	**r = 0.17**	r = 0.10
	p = 0.315	**p<0.001**	p = 0.212	**p = 0.001**	**p<0.001**	**p = 0.040**	p = 0.092
**GGT**	r = 0.04	**r = 0.36**	**r = 0.17**	r = 0.04	**r = 0.18**	**r = 0.34**	**r = 0.17**
	p = 0.450	**p<0.001**	**p = 0.003**	p = 0.515	**p = 0.002**	**p<0.001**	**p = 0.004**

Abbreviations: Circ: circumference; BMI: body mass index; HOMA-IR: HOMA-Index Resistance; Tg: triglycerides; AHI: apnea-hypopnea index; ALT: alanine aminotransferase; AST: aspartate aminotransferase; GGT: gammaglutamyl transpeptidase.

### Prevalence of obesity related diseases according to neck circumference

**Fig 2:**
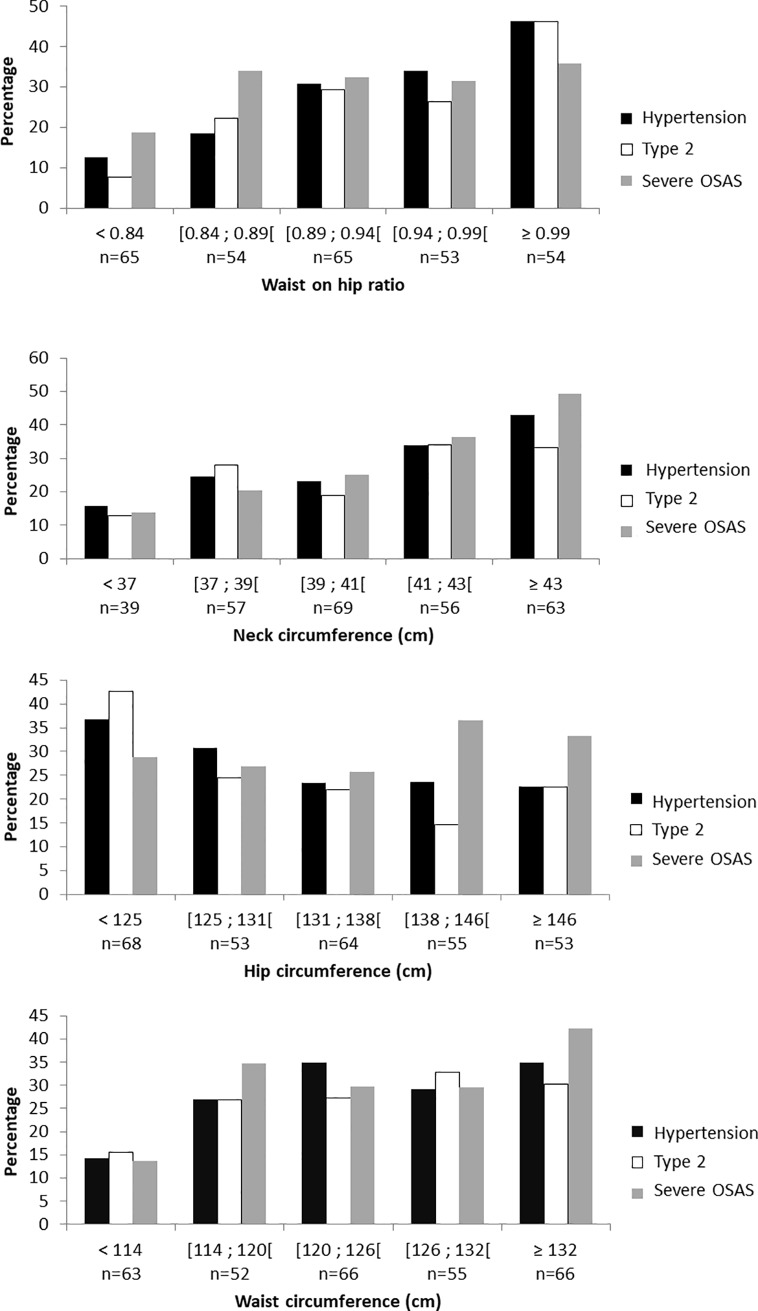
Prevalence of obesity related diseases according to quintiles of anthropometric markers. Abbreviation: OSAS: Obstructive sleep apnea syndrome.

We reported the prevalence of hypertension, type 2 diabetes and severe OSAS according to the different anthropometric markers, divided into quintiles (Fig 2). The prevalence of hypertension progressively increased from 16 to 43%, type 2 diabetes from 13 to 33% and severe OSAS from 14 to 49% across increasing neck circumference quintiles.

## Discussion

The present work studied women with class II or class III obesity who were volunteering for bariatric surgery. The results demonstrated that waist-to-hip ratio was the only anthropometric marker that was unrelated to BMI. The other markers were all collinear with BMI, although neck circumference was less strongly related than waist and hip circumferences. In addition, neck circumference was collinear with markers of central obesity (waist circumference and waist-to-hip ratio) but not with a marker of subcutaneous adiposity (hip circumference).

This study therefore showed that neck circumference was as valid as waist circumference and waist-to-hip ratio for the identification of women with a high cardiometabolic risk profile. Moreover, it is much easier to measure in routine clinical practice in women with severe obesity.

### Reliability of anthropometric markers in the context of severe obesity

In the present work, hip circumference, a marker of subcutaneous adiposity, was not correlated with any metabolic markers and was inversely associated with type 2 diabetes. Previous studies have shown that hip circumference was inversely associated with the risk of incident type 2 diabetes in men and women [18]. Subcutaneous adipose tissue, measured by abdominal computed tomography, was also found to be inversely correlated with type 2 diabetes [4]. An increase in hip circumference reflects an expansion in low body subcutaneous adipose tissue. This subcutaneous fat depot is diametrically opposed to visceral fat. In epidemiological studies, it has been associated with a lower cardiac risk factor burden [19] and lower risk of incident cardiovascular diseases [20] and cancer [21]. This low body subcutaneous fat may impart a protective effect by acting as a metabolic buffer to the influx of dietary lipids and protecting other tissues from the lipotoxicity caused by lipid overflow and ectopic fat deposition [5]. In addition, human obese visceral and subcutaneous adipose tissues secretomes are clearly distinct. Visceral fat secretome leads to a low-grade inflammation profile whereas subcutaneous secretome favors insulin sensitivity [22].

Waist circumference has been shown to be a less reliable marker of metabolic abnormalities in men [23] and women [24] with severe obesity, compared to those with lower or normal BMI scores. This could be attributed to the contribution of abdominal subcutaneous adipose tissue, which appears to be protective for metabolic conditions [25]. However, other studies have also found the well-known relationship between waist circumference and cardiometabolic risk profile in patients with severe obesity [26, 27]. The present work confirmed that in women with severe obesity, waist circumference was associated with hypertension, type 2 diabetes and OSAS, as well as with HOMA-IR and Log TG/HDL, both markers of insulin resistance.

Waist-to-hip ratio and waist circumference, were associated with an increased risk of cardiovascular mortality in women from the Nurses' Health Study prospective cohort [28], as well as in other cohorts of women [29]. In the present study, waist-to-hip ratio in women with severe obesity was a reliable marker for the identification of hypertension, type 2 diabetes (but not OSAS) and other cardiometabolic risk markers. The stronger reliability of this measure over waist circumference alone could be related to the neutralization of abdominal subcutaneous adiposity by the hip circumference denominator, which adjusts waist circumference for subcutaneous adiposity [30]. For the same reason, waist-to-hip ratio is unaffected by increases in BMI, across large ranges of BMI, as confirmed by the present results. However, these results need to be considered according to the ease and accuracy of the physical measurements. It is common clinical experience that waist and hip measurements are subject to errors in patients with severe obesity. This was underlined in our cohort by the outlier data for the patient who had a very high waist-to-hip ratio (1.99) because of a very large abdominal apron. Neck circumference could therefore be a clinically relevant anthropometric marker of central obesity in patients with severe obesity.

### Previous evidence regarding neck circumference

Neck circumference was first identified as a risk marker for OSAS through its use as an index of upper airway restriction by fat deposits in the neck [31]. It has been identified as a relevant tool for clinical screening for OSAS through the STOP-BANG questionnaire [32], which was found to be the most sensitive for the detection of OSAS [8]. In addition, neck circumference was found to be correlated with the visceral adipose tissue, measured by computed tomography in 3307 men (r = 0.63, p<0.001) and women (r = 0.74, p<0.001) in the Framingham health study [33]. Neck circumference was found to be linked with cardiometabolic risk factors after correction for BMI and visceral fat. In other cross-sectional studies, neck circumference was also associated with atherogenic dyslipidemia [34], inflammatory markers (PAI-1) [35], insulin resistance [36], coronary artery disease [37], carotid wall intima-media thickness [38] and NAFLD [39, 40].

Prospective studies have shown that changes in neck circumference are associated with changes in blood pressure [41] and incident type 2 diabetes [42, 43]. Finally, in a cohort of 12,151 patients with a high risk of cardiovascular disease, higher values of neck circumference predicted a higher incidence of fatal and non-fatal cardiovascular events [44].

Thus, neck circumference appears to be a relevant anthropometric marker for the identification of patients with a high cardiometabolic risk associated with central adiposity. Only two previous studies have addressed the question of the relevance of this marker in the context of severe obesity. Assyov et al. [45] found that neck circumference was superior to waist circumference for the identification of a high-risk cardiometabolic profile in 205 men and women with a mean BMI of 36.9 (6.2) kg/m^2^. A cut-off neck circumference value of 37 cm in women was 74% sensitive and 62% specific to identify a risk of type 2 diabetes. Cizza et al. [46] found that neck circumference was associated with metabolic syndrome and OSAS in 102 men and women with a mean BMI of 38.6 (6.5) kg/m^2^, who were identified as short sleepers (<6.5h/night).

Finally, we assessed whether waist-to-height and neck-to-height ratios yielded better results than waist and neck circumferences based on the hypothesis that dividing by height would reduce the variability. Waist-to-height ratio was previously used as a marker of central adiposity in childhood obesity [47]. We found similar results for waist-to-height ratio as for waist circumference, as previously demonstrated in adult women [48]. Accordingly, neck-to-height ratio was previously used in children [49] and adults [50], mainly to screen for sleep apnea. Similarly to our results, neck-to-height ratio did not improve the association observed with neck circumference.

### Study strengths and limitations

The present study sought the easiest and most clinically relevant anthropometric marker for the identification of a high-risk profile for cardiometabolic diseases in women with severe obesity. The study sample was large and the cardiometabolic characteristics of the women were well-characterized. The results are limited by the cross-sectional design of the study, thus it was not possible to determine if anthropometric markers were good predictors of incident metabolic diseases and cardiovascular events in this population, as has been demonstrated in subjects with lower BMIs. The sex ratio of obese patients volunteering for bariatric surgery is largely in favor of women (76% in our cohort). Thus, too few men were included in our cohort to allow the same analyses to be performed in men. However, it would be of interest to study whether the present results are extendable to men in future work. Finally, the population studied was mainly Caucasian thus the results cannot be extrapolated to other ethnicities.

## Conclusion

The present work showed that waist-to-hip ratio and neck circumference have low collinearity with BMI in women with severe obesity. Both markers demonstrated significant associations with prevalent hypertension and type 2 diabetes. Neck circumference was also linked to OSAS. Both were collinear with all other markers of cardiometabolic risk, in particular functional liver tests. Because of the ease of measurement of neck circumference in routine clinical practice, we propose neck circumference to be the best anthropometric marker in women with severe obesity. Further investigation regarding the ability of neck circumference to predict incident cardiometabolic diseases in this population should be carried out. Future studies should evaluate whether a reduction in neck circumference after bariatric surgery is a good marker of cardiometabolic improvement.

## Supporting information

S1 Database (XLS)
